# Lycorine induces cell-cycle arrest in the G0/G1 phase in K562 cells via HDAC inhibition

**DOI:** 10.1186/1475-2867-12-49

**Published:** 2012-11-23

**Authors:** Lv Li, Hong-Juan Dai, Mao Ye, Shu-Ling Wang, Xiao-Juan Xiao, Jie Zheng, Hui-Yong Chen, Yu-hao Luo, Jing Liu

**Affiliations:** 1 Molecular Biology Research Center, School of Biological Science and Technology, Central South University, Changsha, Hunan 410078, China; 2 Molecular Science and Biomedicine Laboratory, State Key Laboratory for Chemo/Biosensing and Chemometrics, College of Biology, Hunan University, Changsha 410082, China; 3 Department of Pharmacology, School of Pharmaceutical Science, Central South University, Changsha, Hunan, 410078, China

**Keywords:** Lycorine, K562 cell line, HDAC inhibition, G0/G1 phase arrest

## Abstract

**Background:**

Lycorine, a natural alkaloid extracted from *Amaryllidaceae*, has shown various pharmacological effects. Recent studies have focused on the potential antitumor activity of lycorine. In our previous study, we found that lycorine decrease the cell viability of leukemia HL-60 cells and multiple myeloma KM3 cells and induces cell apoptosis. However, the effect and molecular mechanism of lycorine on human chronic myelocytic leukemia cells has yet to be determined.

**Methods:**

Human chronic myelocytic leukemia cells K562 were treated with lycorine. Cell viability was monitored using the method of CCK-8. The histone deacetylase (HDAC) enzymatic activity was detected by HDAC colorimetric assay, and the cell cycle was analyzed by flow cytometry. The expression of cell-cycle related proteins were identified using Western blot.

**Results:**

In the present study, we further revealed that lycorine can inhibit the proliferation of K562 cells. Analysis of HDAC activity showed that lycroine decreases HDAC enzymatic activities in K562 cells in a dose-dependent manner. Inhibition of HDAC activity has been associated with cell-cycle arrest and growth inhibition. We evaluated the cell cycle distribution after lycorine treatment and found that lycorine causes cell-cycle arrest in the G0/G1 phase. To investigate the mechanism behind this cell cycle arrest, G1-related proteins were assayed by Western blot. After lycorine treatment, cyclin D1 and cyclin-dependent kinase 4 expressions were inhibited and retinoblastoma protein phosphorylation was reduced. Lycorine treatment also significantly upregulated the expression of p53 and its target gene product, p21.

**Conclusions:**

These results suggest that inhibition of HDAC activity is responsible for at least part of the induction of cell-cycle arrest in the G0/G1 phase by lycorine and provide a mechanistic framework for further exploring the use of lycorine as a novel antitumor agent.

## Background

Leukemia is a type of fatal hematological malignancy. Human chronic myelocytic leukemia (CML), a common type of leukemia, is a myeloproliferative disorder characterized by increased proliferation of granulocytic cell lines with loss capacity to differentiate. CML originates from a constitutive activation of Bcr-Abl tyrosine kinase, which develops from Philadelphia chromosome translocation. Imatinib mesylate (Glivec), a selective inhibitor of Bcr-Abl, was developed as the first molecule-targeted anticancer drug to treat CML patients. However, many patients report developing resistance to Glivec due to mutations in the Abl kinase domain [1,2]. Considering the difficulties inherent in the current CML therapy, the discovery and development new treatment approaches for CML treatment remains an urgent necessity.

Histone acetylation and deacetylation regulate the chromatin structure and gene activation. Histone acetylation is catalyzed by histone acetyltransferases (HATs) and associated with transcriptional activation, whereas histone deacetylation is mediated by histone deacetylases (HDACs) and correlated with chromatin condensation and transcriptional repression [3]. Both of these processes play crucial roles in various biological functions, including cell growth, differentiation, and apoptosis. Dysregulation of these pathways contributes to human cancer development. Several studies have indicated that HDAC inhibitors, compounds that interfere with the function of HDAC, exhibit antitumor activity against various tumor cells by blocking cell cycle progression and inducing apoptosis. Sodium butyrate, an HDAC inhibitor, can suppress breast cancer cell proliferation by blocking the G1/S phase of the cell cycle and activating the apoptosis pathway [4]. Two HDAC inhibitors, suberoylanilide hydroxamic acid (Vorinostat) and romidepsin (Depsipeptide, FK228), were recently approved by the U.S. Food and Drug Administration (USA) for the treatment of cutaneous T-cell lymphoma [5].

Lycorine, a natural alkaloid extracted from *Amaryllidaceae*, has shown various pharmacological effects, such as anti-inflammatory activities, anti-malarial properties, emetic actions, anti-virus effects, and so on [6,7]. Recent studies have focused on the potential antitumor activity of lycorine. Lycorine can reportedly inhibit the growth of multiple tumor cells that are naturally resistant to pro-apoptotic stimuli, such as glioblastoma, melanoma, non-small-cell-lung cancers, and metastatic cancers, among others. Furthermore, lycorine provides excellent in vivo antitumor activity against the B16F10 melanoma model [8]. In our previous study, we found that lycorine decreases the survival rate of and induces apoptosis in HL-60 acute myeloid leukemia cells and the multiple myeloma cell line KM3. The mechanisms of the induced apoptosis were mediated by stimulating the caspase pathway and increasing the Bax: Bcl-2 ratio through downregulation of Bcl-2 expression [9,10]. Lycorine also exhibits significantly higher anti-proliferative activities in tumor cells than in non-tumor cell lines [11].

In this study, we further reveal that lycorine can inhibit proliferation of the human CML cell line K562. Analysis of HDAC activity shows that lycroine decreases HDAC enzymatic activities in K562 cells in a dose-dependent manner. To determine the effect of HDAC inhibition, we evaluate the cell cycle distribution after lycorine treatment. We show that lycorine inhibits the proliferation of K562 cells through G0/G1 phase arrest, which is mediated by the regulation of G1-related proteins. After lycorine treatment, cyclin D1 and cyclin-dependent kinase 4 (CDK4) expressions are inhibited and retinoblastoma protein (pRB) phosphorylation is reduced. Lycorine treatment also significantly upregulates the expression of p53 and its target gene product, p21. These results suggest that inhibition of HDAC activity is responsible for at least part of the induction of G1 cell-cycle arrest of K562 cells by lycorine.

## Results

### Lycorine inhibits the proliferation of K562 cells

**Figure 1:**
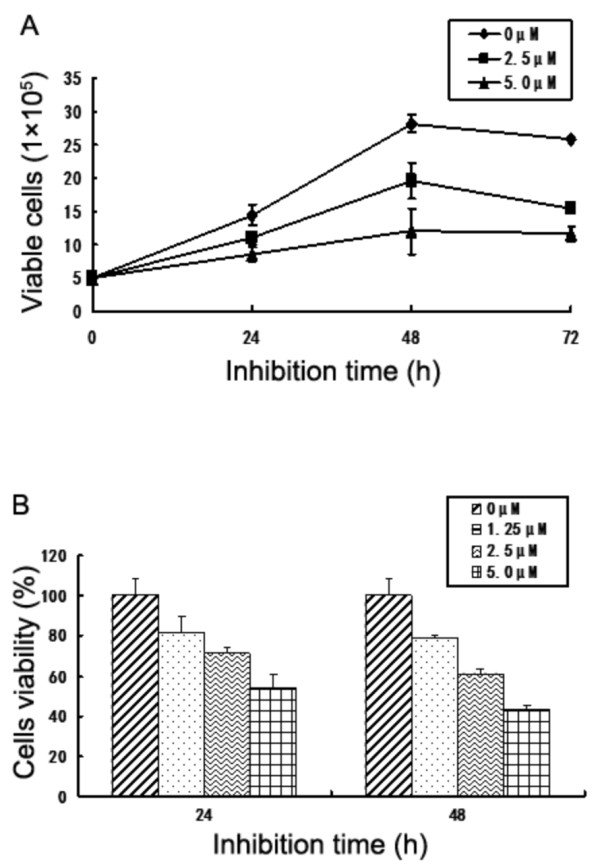
**Lycorine decreases K562 cell growth.** (**A**) Effect of lycorine on the proliferation of K562 cells. Cells were cultivated in RPMI 1640 medium with indicated concentrations of lycorine for 24, 48, and 72 h. The cell density was counted by manual cell counting. (**B**) Effect of lycorine on the survival rate of K562 cells. Cells were cultivated in RPMI 1640 medium with indicated concentrations of lycorine for 24 and 48 h. The cell survival rate was calculated by CCK-8 method. Each value represents the mean ± S.D. of three independent experiments.

To determine the effect of lycorine on the growth of CML cells, K562 cells were treated with lycorine at various concentrations and examined by manual cell counting every 24 h for 72 h. Compared with the control group, the cells density of the group treated with 5.0 μM lycorine increased very slightly from 24 h to 72 h, which indicates that lycorine significantly inhibits the growth of K562 cells (Figure 1A). CCK-8 assays showed that the viability of K562 cells exposed to various concentrations of lycorine (1.25, 2.5, and 5.0 μM) decreased from 82% to 54% after 24 h and from 80% to 42% after 48 h, which reveals that lycorine inhibits the proliferation of K562 cells in a dose-dependent manner (Figure 1B).

### Lycorine inhibits the enzymatic activity of HDACs

**Figure 2:**
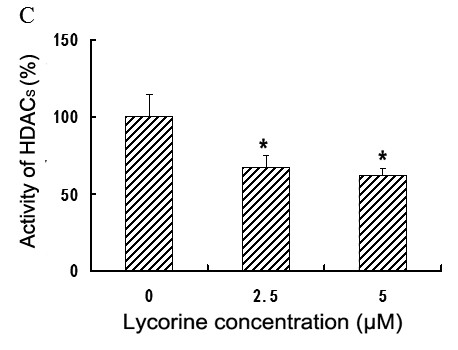
**Effects of lycorine on the activity of HDACs in K562 cells.** Briefly, nuclear proteins were extracted from K562 cells treated with different concentrations of lycorine (2.5 or 5.0 μM) or without lycorine for 24 h. About 50 μg of nuclear protein from each group was added to a 96-well tissue culture plate and HDAC enzymatic activities in the cell nucleus was determined according to the HDAC colorimetric assay kit instructions. Values are expressed as the percentage of HDAC activities relative to untreated cell extracts. Results are presented as mean ± S.D. (n = 3, three independent experiments). Asterisks (*) indicate significant difference (*P* < 0.05) compared with the control group.

Histone acetylation and deacetylation regulate the chromatin structure and gene transcription. Dysregulation of their function has been associated with human cancer development. Recent studies have utilized HDAC as a potential target for the development of new therapeutic agents [3]. To determine the effect of lycorine on HDACs, we detected the expression of HDAC1 and HDAC3 proteins in K562 cells after lycorine treatment. We found that lycorine did not change the expression of HDAC1 and HDAC3 proteins (data not shown), whereas lycorine-treated K562 cells significantly showed decreased HDAC activity of 24 h after treatment (Figure 2). These results reveal that lycroine directly inhibits HDAC enzymatic activities but does not affect HDAC expression in K562 cells.

### Lycorine induces cell cycle arrest in the G0/G1 phase

**Figure 3:**
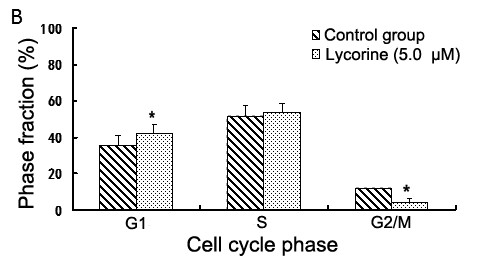
**Lycorine increases the proportion of G0/G1 phase cells and decreases the proportion of G2/M phase cells.** After the K562 cells were cultivated with lycorine (5 μM), the percentage of cells in the G1 phase increased gradually from 35.9% to 41.9% after 24 h and the percentage of cells in the S phase increased slightly from 51.7% to 53.6%. Asterisks (*) indicate significant difference (*P* < 0.05) compared with the control group.

Inhibition of HDAC activity has been associated with cell-cycle arrest and growth inhibition. Thus, we determined whether or not lycorine can interfere with cell cycle progression by flow cytometry. After K562 cells were treated with 5 μM lycorine, the percentage of cells in the G0/G1 phase increased significantly from 35.9% to 41.9% while S-phase cells showed only a slight increased. The percentage of G2/M-phase cells decreased from 12.3% in the untreated group to 4.44% in the treated group (Figure 3). This finding indicates that cell cycle distribution was blocked significantly in the G0/G1 phase when K562 cells are treated with lycorine.

### Lycorine regulates the expression of cell cycle-related proteins in K562 cells

**Figure 4:**
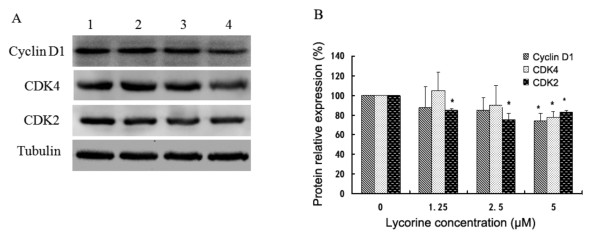
**Effects of lycorine on expression of cyclin D1, CDK4 and CDK2 in K562 cells.** Tubulin was used for normalization and verification of protein loading in Western blot analysis. Lanes 1, 2, 3, and 4 represent K562 cells treated with 0, 1.25, 2.5, and 5.0 μM lycorine, respectively. Results are presented as mean ± S.D. (n = 3, three independent experiments). Asterisks (*) indicate significant difference (*P* < 0.05) compared with the control group.

To reveal the molecular mechanism of cell-cycle arrest in the G0/G1 phase, we investigated whether or not the effects induced by lycorine were associated with the level of G1-S transition-related proteins. After treating K562 cells with various concentrations of lycorine, we observed a dose-dependent decrease in cyclin D1 levels. The decrease in cyclin D1 expression observed in lycorine-treated cells was accompanied by a reduction in the amount of CDK4 and CDK2 (Figure 4A and 4B). By contrast, the expression patterns of cyclin E and CDK6 were not significantly altered after treatment with lycorine (data not shown).

**Figure 5:**
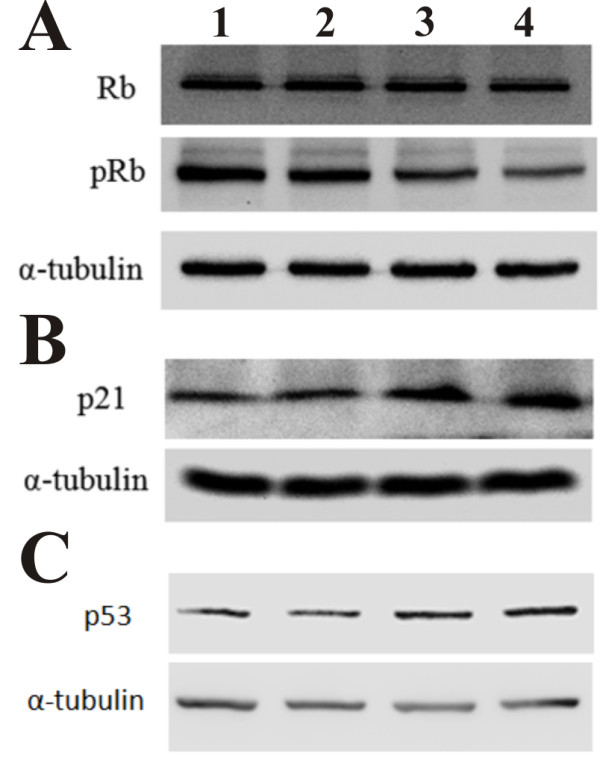
**Effects of lycorine on expression of p21, p53 and pRB, and phosphorylation of pRB in K562 cells.** Lanes 1, 2, 3, and 4 represent K562 cells treated with 0, 1.25, 2.5, and 5.0 μM lycorine, respectively. α-tubulin was used for normalization and verification of protein loading in Western blot analysis. Results are presented as mean ± S.D. (n = 3, three independent experiments).

To examine the effect of lycorine on the phosphorylation of pRB, K562 cells were treated with different concentrations of lycorine, after which proteins were detected using antibodies specific to the total pRB and phosphorylated pRB. Results show that the expression of total pRB remains almost unchanged but the level of phosphorylated pRB decreases significantly in a dose-dependent manner (Figure 5).

p21, as a CDK inhibitor, can interfere with cancer cell cycle and affect cell proliferation [12]. p21 binds to and inhibits the activity of cyclin E-CDK2 complexes, which cause pRB hypophosphorylation and cell-cycle arrest at the G1-S transition. We further explored the expression of p21 at the protein level and found that lycorine could induce a dose-dependent increase in p21 in K562 cells (Figure 5). Consistent with the change in p21, the expression of p53 protein was also elevated, which suggests that lycorine induces the expression of p21 in a p53-dependent manner in K562 cells (Figure 5).

## Discussion

HATs and HDACs regulate the chromatin structure and gene transcription. Their dynamic balance plays a crucial role in various biological functions, including cell proliferation and death. Their dysregulation has been related to the development and progression of various cancers, including forms of myeloid leukemia [13,14]. Recent studies have utilized HDACs as a promising target enzyme in anticancer drug development. Several studies have shown that HDAC inhibitors can induce differentiation of tumor cells, arrest the cell cycle at the G0/G1 phase, and activate the cell apoptosis gene. Normal cells are relatively resistant to HDAC inhibitor-induced cell death [15,16]. The results of our study reveal that lycorine inhibits the activity of HDACs but does not affect their expression in K562 cells, which indicates that lycorine is a promising potential therapy agent in CML. However, the detailed molecular mechanism behind the inhibition of HDAC enzymatic activity by lycorine must be investigated further.

Several studies have shown that inhibitors of HDAC block cell cycle progression at the G0/G1 or G2/M phase [17] depending on the cell type and type of drugs. Similar to the effect of HDAC inhibitors in other tumor types, lycorine inhibits cell cycle progression and induces cell-cycle arrest in the G0/G1 phase in K562 cells. Progress in the eukaryotic cell cycle is driven by protein kinase complexes consisting of a cyclin and a CDK. During G1-phase progression, the complexes cyclin D-CDK4, cyclin D-CDK6, and cyclin E-CDK2 are activated and move the cell cycle from the G1 phase to the S phase. We found that cyclin D1, CDK4 and CDK2 are significantly downregulated in K562 cells after lycorine treatment. By contrast, the expression patterns of cyclin E, CDK2, and CDK6 were not significantly altered after lycorine treatment. This finding suggests that inhibition of cyclin D1 and CDK4 expression is involved in lycorine-induced G0/G1 arrest in K562 cells.

During G1-phase progression, pRB is phosphorylated by cyclin D-CDK4, CDK6, and cyclin E-CDK2 complexes. Hyperphosphorylation of pRB inactivates its function and dissociates the E2F transcription factor from pRB, which is critical to progression to the S phase. We found that, the expression level of pRB remains constant in lycorine-treated K562 cells, whereas the level of phosphorylated pRB decreases significantly, indicating that lycorine can suppress pRB phosphorylation. Thus, hypophosphorylated pRB combines E2Fs more tightly, induces cell cycle arrest, and prevents proliferation.

CDK activity is regulated negatively by a group of proteins called CDK inhibitors, including the protein p21 WAF1/CIP1 (p21). p21 protein binds to and inhibits the activity of cyclin E-CDK2 complexes, which causes pRB hypophosphorylation and cell cycle arrest in the G1–S transition. Expression of the p21 gene is tightly controlled by the tumor suppressor p53. The results of our study show that lycorine treatment significantly upregulates the expression of p21 in K562 cells. Consistent with the change in p21, the expression of p53 protein is also elevated, which suggests that lycorine may induce the expression of p21 in a p53-dependent manner in K562 cells.

## Conclusions

In summary, our data show that lycorine can inhibit proliferation of the human CML cell line K562 through G0/G1 phase arrest, which is mediated by the regulation of G1-related protein. Meanwhile, the inhibition of HDAC enzymatic activity is involved in the effect of lycorine on K562 cells. Further in-depth in vivo studies are presently under investigation in our laboratory.

## Materials and methods

### Cell culture and drugs

The human CML cell line K562 was purchased from American Type Culture Collection and cultivated in RPMI 1640 medium (Gibco) supplemented with 10% heat-inactivated fetal bovine serum (Gibco), 100 U/mL streptomycin, and 100 U/mL penicillin at 37°C in a humidified atmosphere with 5% CO_2_. Cells were diluted at a ratio of 1:3 every 1 d to 2 d. Lycorine (Sigma) was dissolved at 0.034 M in dimethyl sulfoxide (DMSO; Sigma) as a stock solution and diluted in serum-free RPMI 1640 medium just before use. The maximum final concentration of DMSO in medium was less than 0.02%.

### Cell counting

To examine the anti-proliferative effect of lycorine, growth curves were protracted by manual cell counting. Exponentially growing K562 cells treated with different concentrations of lycorine (2.5 or 5.0 μM) or without lycorine were cultivated at 5 × 10^5^ cells/mL in a culture flask (BioCoat). After appropriate culture, viable cells were counted manually and continuously for up to 3 d.

### Cell viability and cytotoxicity assay (CCK-8)

Cell viability and cytotoxicity were measured with 2-(2-methoxy-4-nitrophenyl)-3-(4-nitrophenyl)-5-(2,4-disulfophenyl)-2H tetrazolium monosodium salt (CCK-8) assay as described previously. Briefly, exponentially growing K562 cells treated with various concentrations of lycorine (1.25, 2.5, or 5.0 μM) or without lycorine were cultivated at 1.25 × 10^4^ cells/well in a 96-well tissue culture plate (BioCoat) at a total volume of 100 μL per well. After cells were incubated for 24 and 48 h, 10 μL of CCK-8 solution (Beyotime) was added to each well and incubation of cells was performed for another 4 h at 37°C. The relative cell viability was determined by scanning with an ELISA reader with a 450 nm filter and calculated by CCK-8 assay.

### Detection of HDAC activities

A HDAC colorimetric assay kit (Biovision) was applied to determine HDAC enzymatic activities in the cell nucleus according to the manufacturer’s instructions. Briefly, proteins were extracted from K562 cells treated with different concentrations of lycorine (2.5 or 5.0 μM) or without lycorine for 24 h using a nuclear and cytoplasmic protein extraction kit (Beyotime) according to manufacturer recommendations. About 50 μg of nuclear protein from each group was added to a 96-well tissue culture plate (BioCoat) at a final volume of 100 μL per well. After incubation, HDAC activities were measured by scanning with an ELISA reader with a 450 nm filter. Values were expressed as the percentage of HDAC activities relative to the untreated cell extract.

### Flow cytometry

Flow cytometry was used to detect the cell cycle distribution and quantitatively measure the apoptotic rate. After K562 cells treated with lycorine (5.0 μM) or without lycorine were cultivated at 5 × 10^5^ cells/mL in each culture flask (BioCoat) for 24 h, 1 × 10^6^ cells were harvested and washed with PBS. The cells were then fixed with ice-cold 70% ethanol at −20°C overnight. The next day, the cells were washed with PBS, stained with 50 mg/mL propidium iodide (Sigma), and dissolved in 100 mg/L RNase A (Sigma). The sub-G1 peak (apoptosis percentage) and cell cycle distribution were measured with Cytomic FC 500 (Beckman Coulter) and analyzed using Modifit LT software.

### Western blot analysis

Exponentially growing K562 cells treated with various concentrations of lycorine (1.25, 2.5, or 5.0 μM) or without lycorine were cultivated at 5 × 10^5^ cells/mL in several culture flasks (BioCoat). After 24 h of culture, the cells were pelleted by centrifugation, washed three times with PBS, resuspended in 100 μL of RIPA lysis buffer (20 mM Tris–HCl, pH 7.5; 150 mM NaCl, 1 mM EDTA, 1% Nonidet P40, 0.5% deoxycholate, 0.1% SDS, 5 mM NaF, and 0.5% cocktail), and centrifuged at 13000 rpm and 4°C for 15 min to collect the supernatant. The supernatant protein concentration was measured using a bicinchoninic acid protein assay kit (Thermo Scientific). Equal amounts of protein (50 μg) from each group were electrophoresed for 2 h on 10% sodium dodecyl sulfate-polyacrylamide gels and then transferred to a PVDF membrane (Millipore) using an electroblotter for 100 min at 4°C. Membranes were blocked in PBS with 0.1% Tween 20 (PBST) containing 5% non-fat dried milk power for 1 h. An antibody (Abcam) raised against α-tubulin (1:20000), an antibody (Bioworld) raised against pRB (1:2000), an antibody (Bioss) raised against p21 an antibody (Cell Signaling) raised against phosphorylated pRB (1:3000), and antibodies (Santa Cruz Biotech) raised against p53 (1:3000), cyclin D1 (1:200), CDK4 (1:200), and CDK2 (1:500) were diluted in PBST containing 5% non-fat milk and membranes were incubated overnight at 4°C. After washing four times with PBST for 10 min each time, the blot was incubated with anti-mouse or anti-rabbit IgG conjugated with horseradish peroxidase (Millipore, 1:5000 dilution in PBST containing 5% non-fat milk) for 1 h at room temperature. After washing three times with PBST for 10 min each time, the blots were developed with a chemiluninescene detection kit (ECL; Millipore), and the optical density of each band was quantified by densitometric scanning.

### Statistical analysis

The statistical difference between groups was determined by AVOVA and Tukey’s studentized range test. Differences among groups were considered statistically different at *P* < 0.05.

## Misc

Lv Li and Hong-Juan Dai contributed equally to this work.

## Competing interests

The authors declare that they have no competing interests.

## Authors’ contributions

LL, HJD, SLW, XJX, JZ, HYC and YHL performed experiments and summarized the data; MY and JLdesigned experiments; LL, MY and JL wrote the paper. All authors have read and approved the final manuscript.
